# Efficacy and Safety of Once-Weekly Semaglutide for the Treatment of Type 2 Diabetes: A Systematic Review and Meta-Analysis of Randomized Controlled Trials

**DOI:** 10.3389/fphar.2018.00576

**Published:** 2018-06-04

**Authors:** Fang-Hong Shi, Hao Li, Min Cui, Zai-Li Zhang, Zhi-Chun Gu, Xiao-Yan Liu

**Affiliations:** ^1^Department of Pharmacy, Renji Hospital, School of Medicine, Shanghai Jiaotong University, Shanghai, China; ^2^Department of Pharmacy, Shanghai Children's Medical Center, School of Medicine, Shanghai Jiaotong University, Shanghai, China

**Keywords:** type 2 diabetes, glucagon-like peptide-1 receptor agonists, semaglutide, randomized controlled trials, meta-analysis

## Abstract

**Background:** Semaglutide, a newly once-weekly glucagon like peptide-1 (GLP-1) receptor agonist, has showed a favorable effect on glycaemic control and weight reduction in type 2 diabetes mellitus (T2DM). This meta-analysis was conducted to evaluate the clinical efficacy and safety of semaglutide in T2DM.

**Methods:** A comprehensive searching was performed for Phase III randomized controlled trials (RCTs) which reported the efficacy and safety data of semaglutide and other therapies. The efficacy data expressed as weight mean difference (WMD) and the safety data expressed as risk ratios (RRs) were calculated by employing random-effects model. Heterogeneity was assessed through I^2^ test, and subgroup analyses were performed by different control groups, dosage of semaglutide, and durations of follow up.

**Results:** 9 RCTs including 9,773 subjects met the inclusion criteria. For efficacy, compared with other therapies, semaglutide resulted in a significant reduction in glycosylated hemoglobin (weight mean difference, WMD: −0.93%, 95% CI: −1.24 to −0.62, *P* < 0.001), fasting plasma glucose (WMD: −1.15 mmol/L, 95% CI: −1.67 to −0.63, *P* < 0.001), mean self-monitoring of plasma glucose (WMD: −1.19 mmol/L, 95% CI: −1.68 to −0.70, *P* < 0.001), body weight (WMD: –3.47 kg, 95% CI: −3.96 to −2.98, *P* < 0.001), body mass index (WMD: –1.25 kg/m^2^, 95% CI: −1.45 to −1.04, *P* < 0.001), systolic blood pressure (WMD: −2.55 mmHg, 95% CI: −3.22 to −1.88, *P* < 0.001), with the exception of negative result of diastolic blood pressure (WMD: −0.29 mmHg, 95% CI: −0.65 to 0.07, *P* = 0.113) and increased impact on pulse rate (WMD: −2.21, 95% CI: 1.54 to 2.88, *P* < 0.001). The results were consistent across the key subgroups. For safety, semaglutide did not increase the risk of any adverse events, hypoglycemia and pancreatitis, but induced a higher risk of gastrointestinal disorders when compared with other therapies (RR: 1.98, 95%CI: 1.49 to 2.62, *P* < 0.001).

**Conclusion:** Semaglutide was effective and acceptable in patients with T2DM except for a high risk of gastrointestinal disorders. The capacity of glycaemic and body weight control of semaglutide appeared more effective than other add-on therapies including other GLP-1 receptor agonists of exenatide release and dulaglutide.

## Introduction

Type 2 diabetes mellitus (T2DM) is a complex and progressive disease due to a progressive loss of β-cell insulin secretion frequently on the basis of insulin resistance that manifests clinically as hyperglycemia (Inzucchi et al., 2015). Despite various medications are now available for the treatment of T2DM, it remains a challenge to select anti-diabetic agents that come with a good balance between efficacy and safety.

Metformin is generally recommended as the first-line therapeutic agent in T2DMs with lifestyle changes according to the American Diabetes Association (ADA) and European Association for the Study of Diabetes (ADA/EASD).(Inzucchi et al., 2012).

When lifestyle changes and maximally tolerable dose of metformin fail to control hyperglycemia, other anti-hyperglycemic drugs are necessary to better control of glucose including oral antihyperglycemic drugs (sulfonylureas, thiazolidinedione, DPP4 inhibitors, alpha glucosidase inhibitors etc.) and injectable anti-hyperglycemic drugs (insulin, Glucagon-like peptide-1 receptor agonists etc.) (Koro et al., 2004; Doggrell, 2018). Of note, the effect of long-term glycemic control may not be maintained owing to gradually declined beta cell function or subsequently increased cardiovascular risk (Turner et al., 1999). Therefore, the selection of anti-hyperglycemic drugs balancing the efficacy and safety is needed.

Glucagon-like peptide-1 receptor agonists (GLP-1 RAs), a kind of secreted peptide that release from neuroendocrine intestinal L-cells, are recently recommended by American Diabetes Association/European Association for the Study of Diabetes (ADA/EASD) as a second-line treatment when first-line treatment (mainly metformin monotherapy) fails to achieve well controlled glucose. Owing to their efficacy on glycemic control and reduction of body weight and blood pressure (BP), with a low risk of hypoglycemia, GLP-1RAs are extensively used in diabetes patients (Inzucchi et al., 2012; Dugan, 2017).

Semaglutide, a newly subcutaneous and long acting GLP-1 RA with 94% structural homology to native GLP-1, has been approved by the United States Food and Drug Administration (FDA) on December 5, 2017, as an adjunct to diet and exercise for the treatment of T2DM (Lau et al., 2015; Dhillon, 2018). Semaglutide has three modified GLP-1 peptides that contains two amino acid substitutions as compared to native GLP-1 (Aib8, Arg34) and derivatized at lysine 26. Semaglutide is similar to liraglutide in structure, but more resistant property than liraglutide by structural modifications, making it less susceptible to degradation by enzyme dipeptidyl peptidase-4 and more albumin affinity (Lau et al., 2015). The molecular modification of semaglutide brings about a long half-life of 165 h, which may represent a preferably once-weekly GLP-1 analog (Kapitza et al., 2015). Although semaglutide has been evaluated in several randomized trials, the overall evaluation of semaglutide is urgent. We thus conducted a systemic review and meta-analysis to present a comprehensive picture on the efficacy and safety of semaglutide in patients with T2DM.

## Materials and methods

### Study design

This meta-analysis was conducted in accordance to the Preferred Reporting Items for Systematic Reviews and Meta-Analyses (PRISMA) guidelines and was conducted following a priori established protocol (PROSPERO: CRD42018084958) (Moher et al., 2010). Ethical approval is not required because this is a systemic review study. A comprehensively systematic search of Medline, Embase, and the Cochrane library was conducted from inception to Feb 24th, 2018 without language restriction. Additionally, unpublished trials were identified from the “ClinicalTrials.gov” website. References of all pertinent articles were further scrutinized to ensure that all relevant studies were identified. For the topic of “type 2 diabetes,” the following key terms were used for searching: “type 2 diabetes” or “type 2 diabetes mellitus”. For the topic of “semaglutide,” we included the following terms: “semaglutide” or “NN9936” or “NN9934” or “NN9935” or “ozempic.” For the topic of “randomized controlled trials (RCTs),” the terms used were: “clinical trial” or “controlled clinical trial” or “randomized controlled trial.” Finally, we used the Boolean operator “AND” to combine three comprehensively searching topics. Two reviewers (Fang-Hong Shi and Hao Li) independently searched the databases to identify all potentially eligible studies, and all disagreements were resolved by consensus or by consulting a third author (Zhi-Chun Gu).

### Inclusion criteria and study selection

All Phase III RCTs assessing the efficacy and safety of semaglutide in T2DM were considered as a potentially eligible paper. The predetermined study inclusion criteria were: (1) RCTs; (2) adult patients had T2DM; (3) compared semaglutide with other therapies (anti-diabetic therapy or placebo); (4) reported the interested efficacy data including estimated treatment difference about glycemic control, weight control, and blood pressure and pulse rate; (5) reported safety data including adverse events (AEs) with varying degrees and AEs occurring in ≥ 5% patients according to predetermined terms or clinical significance; (6) the duration of follow up should be more than 24 weeks.

### Data extraction

Two investigators (Fang-Hong Shi and Hao Li) screened the titles and abstracts of retrieved citations independently to identify potentially eligible trials. Following data were extracted from the eligible trials: first author's name, year of publication, number of study patients, baseline patient characteristics, related efficacy and safety data.

### Quality assessment and bias assessment

Two investigators (Fang-Hong Shi and Hao Li) evaluated the methodological quality of included randomized trials according to the Cochrane Collaboration Risk of Bias Tool, which includes random sequence generation, allocation concealment, masking, incomplete outcome data, selective reporting, and other bias (Higgins et al., 2011). Furthermore, we also assessed the background medication administration and funding sources. Any disagreement was settled by discussing with the third author (Zhi-Chun Gu). Potential publication bias was evaluated by visually inspecting funnel plots as well as quantitative analysis of Begg test and Egger test (Egger et al., 1997).

### Data analysis

The estimates of meta-analysis were derived and presented in forest plots by using STATA version 12.0 (STATA Corporation, College Station, TX, USA) (Jaïs et al., 2008). Continuous variables were expressed as weight mean difference (WMD) with their 95% confidence intervals (95% *CIs*), and dichotomous data were reported as risk ratios (RRs) with 95% *CIs*. The random-effects model was used to calculate the overall estimated effects. Heterogeneity, which measures the percentage of total variation between studies, was tested through the *I*^2^ test (Chen et al., 2017). Subgroup analyses were performed by different control groups (placebo, sitagliptin, insulin glargine, other GLP-1 RAs, and other oral anti-diabetic drug), dosage of semaglutide (0.5 or 1.0 mg weekly) and duration of follow up. We conducted a sensitivity analysis to evaluate the influence of each individual study by omitting one study at a time as well as the impact after removing the placebo-controlled studies. *P* < 0.05 indicated a statistically significant difference.

## Results

### Study evaluation

**Figure 1:**
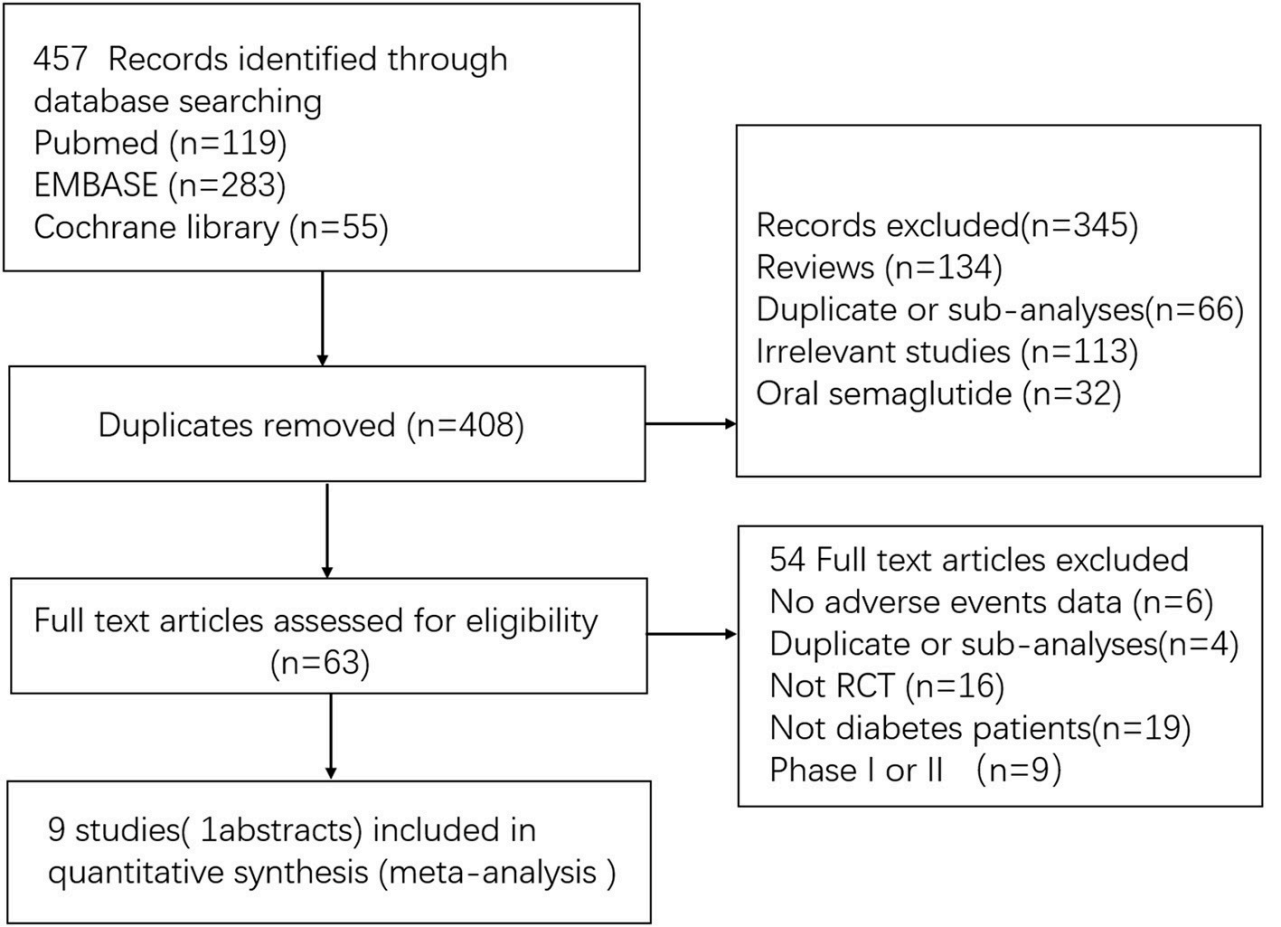
Flow diagram for the selection of eligible randomized controlled trials. RCT indicates randomized controlled trial.

A total of 457 initially relevant publications were identified. Of these, 448 records were excluded through screening title and abstract by different reasons (Among these records, 10 RCTs were excluded which were listed in Table S1). Finally, 9 studies were identified for the final analysis (including 1 abstract) (Figure 1) (Conway et al., 2016; Marso et al., 2016; Ahrén et al., 2017; Aroda et al., 2017; Sorli et al., 2017; Ahmann et al., 2018; Kaku et al., 2018; Pratley et al., 2018; Seino et al., 2018). The characteristics of the included RCTs were represented in Table 1. Publication year varied from 2016 to 2018, and the trial duration ranged from 30 to 104 weeks. In total, 9,773 participants were included, consisting of 5,774 patients in semaglutide group and 3,999 patients in other therapies group. Regarding comparators in included studies, 3 studies (4,550 patients) received placebo, 2 studies (1,533 patients) received sitagliptin, 2 studies (2,008 patients) received other GLP-1 RAs (exenatide release or dulaglutide), 1 study (1,082 patients) received insulin glargine and 1 study (600 patients) received other oral antidiabetic drugs. Among included 9 RCTs, 5 studies involving 3,998 patients were open label studies (Aroda et al., 2017; Ahmann et al., 2018; Kaku et al., 2018; Pratley et al., 2018; Seino et al., 2018). All trials satisfied all bias tool items with the exception of blind method. Thus, the overall quality of included trials was moderate to high (Table S2).

**Table 1:** Demographic and other baseline characteristics.

**Study**	**Sorli C (SUSTAIN1)**			**Ahrén B (SUSTAIN2)**			**Ahmann AJ (SUSTAIN3)**		**Aroda VR (SUSTAIN4)**			**Marso SP (SUSTAIN6)**			**Richard EP (SUSTAIN7)**			**Seino Y (SUSTAIN**™**)**			**Kaku K (SUSTAIN**™**)**		
NCT number	NCT02054897			NCT01930188			NCT01885208		NCT02128932			NCT01720446			NCT02648204			NCT02254291			NCT02207374		
Year	2017			2017			2018		2017			2016			2018			2018			2018		
Follow up (weeks)	30			56			56		30			104			40			30			56		
Background	DE			MET+TZD			OAD		MET±SU			AHA			MET			DE±OAD			DE or OAD		
Variable	SEM	SEM	PLA	SEM	SEM	SIT	SEM	EXER	SEM	SEM	GLA	SEM	SEM	PLA	SEM	SEM	DUL	SEM	SEM	SIT	SEM	SEM	OAD
Dosage (mg)	0.5	1.0	N/A	0.5	1.0	N/A	1.0	2.0	0.5	1.0	N/A	0.5	1.0	N/A	0.5	1.0	N/A	0.5	1.0	N/A	0.5	1.0	N/A
Participants	128	130	129	409	409	407	404	405	362	360	360	826	822	1649	301	300	598	103	102	103	239	241	120
Mean age (year)	54.6	52.7	53.9	54.8	56.0	54.6	56.4	56.7	56.5	56.7	56.2	64.6	64.7	64.6	56.0	55.0	56.0	58.8	58.1	57.9	58.0	58.7	59.2
**HbA1C**																							
%	8.1	8.1	8.0	8.0	8.0	8.2	8.4	8.3	8.1	8.3	8.1	8.7	8.7	8.7	8.3	8.2	8.2	8.2	8.0	8.2	8.0	8.1	8.1
mmol/mol	64.9	65.3	63.4	64.1	64.4	65.8	67.9	67.7	65.4	66.6	65.4	NK	NK	NK	67.5	65.7	66.2	NK	NK	NK	64.4	65.5	65.1
mean duration of diabetes (years)	4.8	3.6	4.1	6.4	6.7	6.6	9.0	9.4	7.8	9.3	8.6	14.3	14.1	13.6	7.7	7.0	7.4	8.0	7.8	8.1	8.1	9.4	9.3
Body weight (kg)	89.8	96.9	89.1	89.9	89.2	89.3	96.2	95.4	93.7	94.0	92.6	91.8	92.9	91.9	96.4	95.6	94.5	67.8	70.8	69.4	71.0	71.7	72.2
BMI (kg/m^2^)	32.5	33.9	32.4	32.4	32.5	32.5	34.0	33.6	33.1	33.0	33.0	32.7	32.9	32.8	33.7	33.6	33.4	25.1	26.1	25.1	26.2	26.4	26.7
eGFR (MDRD; ml/min per 1.73 m^2^)	95.9	100.9	100.2	97.0	97.0	98.0	100.5	100.5	97.9	98.0	99.7	NK	NK	NK	96.0	96.0	96.0	NK	NK	NK	101.4	101.6	102.0
**SEX (%)**																							
Female	53	38	46	49	50	49	46	44	46	49	46	40	37	40	44	46	45	23	27	21	31	28	26
Male	47	62	54	51	50	51	54	56	54	51	54	60	63	60	56	54	55	77	73	79	69	72	74
**ETHNIC ORIGIN (%)**																							
Hispanic or latino	27	35	28	17	16	18	23	26	17	21	22	16	15	15	10	10	13	NK	NK	NK	NK	NK	NK
Not Hispanic or latino	73	65	72	83	84	82	77	74	83	79	78	84	85	85	90	90	87	NK	NK	NK	NK	NK	NK
**RACE (%)**																							
White	65	68	60	68	68	69	84	84	77	78	77	84	84	82	77	78	78	NK	NK	NK	NK	NK	NK
Black or African American	9	8	7	4	6	4	7	7	9	9	9	7	7	7	6	6	6	NK	NK	NK	NK	NK	NK
Asian	20	19	25	26	24	25	2	2	12	11	11	8	7	9	17	16	16	NK	NK	NK	NK	NK	NK

*SUSTAIN5 only abstracts available, thus baseline characteristics cannot be obtained. HbA1C, glycosylated hemoglobin; BMI, body mass index; eGFR, estimated glomerular filtration rate; MDRD, modification of diet in renal disease; SEM, semaglutide; PLA, placebo; EXER, exenatide release; GLA, insulin glargine; DUL, dulaglutide; SIT sitagliptin; OAD, oral antidiabetic drug; AHA, antihyperglycaemic agent; MET, metformin; TZD, thiazolidinediones; SU, sulfonylureas; DE, diet and exercise; N/A, not applicable; NK, not known*.

### Efficacy analysis

#### Glycemic control (glycosylated hemoglobin%, fasting plasma glucose, self-monitoring of plasma glucose and postprandial self-monitoring of plasma glucose)

**Figure 2:**
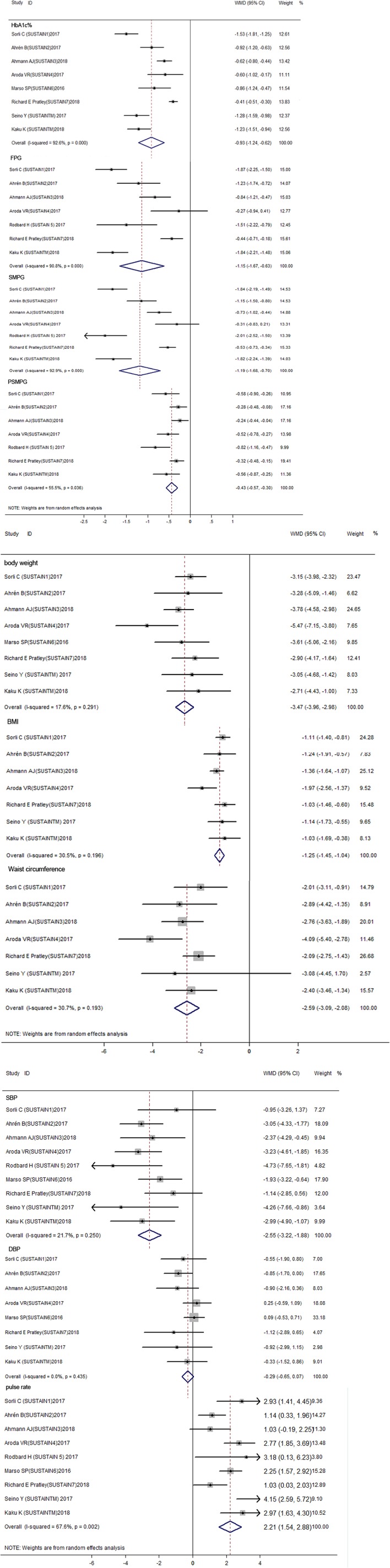
Forest plots for the changes of HbA1C%, FPG, SMPG mean, SMPG Postprandial increment, body weight, BMI, Waist circumference, DBP, SBP, Pulse rate between semaglutide treated and control treated patients with T2DM **(A–C)**. Glycemic control indicators including HbA1C%, FPG, SMPG mean, SMPG Postprandial increment etc. significantly decreased between semaglutide treated and control group (*P* < 0.001) **(A)**. Body weight, BMI, Waist circumference also decreased through semaglutide vs. control group (*P* < 0.001) **(B)**. For blood pressure indicators, SBP and Pulse rate have significantly difference between semaglutide and control group (*P* < 0.001), While difference of DBP is not significant (*P* = 0.113), in which semaglutide decrease SBP but increase pulse rate **(C)**. HbA1C, glycosylated hemoglobin; FPG, fasting plasma glucose; SMPG, self-monitoring of plasma glucose; BMI, body mass index; DBP, diastolic blood pressure; SBP, systolic blood pressure.

Figure 2 summarized the overall efficacy results of semaglutide, and Table S3 presented the corresponding subgroup results. For glycosylated hemoglobin (HbA1c%), the result showed that semaglutide significantly decreased the HbA1c% level when compared with other therapies (WMD: −0.93%, 95% CI: −1.24 to −0.62, *P* < 0.001). As for fasting plasma glucose (FPG), the use of semaglutide was associated with a lower FPG concentration compared with other therapies (WMD: −1.15 mmol/L, 95% CI: −1.67 to −0.63, *P* < 0.001). Regarding self-monitoring of plasma glucose (SMPG), which reflects average level of glycemic control after 7–8 times testing a day, has been recommended in the process of self-management in diabetes patients. The results showed a significantly decrease in SMPG (WMD: −1.19 mmol/L, 95% CI: −1.68 to −0.70, *P* < 0.001) as well as postprandial self-monitoring of plasma glucose (PSMPG) (WMD: −0.43 mmol/L, 95% CI: −0.57 to −0.30) with semaglutide vs. other therapies. The considerable heterogeneity was detected across above outcomes (*I*^2^ = 92.6% for HbA1c%, *I*^2^ = 90.8% for FPG, *I*^2^ = 92.9% for SMPG, and *I*^2^ = 55.5% for PSMPG).

#### Weight control (body weight, body mass index, and waist circumference)

The results of weight control were presented in Figure 2 and Table S3. With regard to body weight, semaglutide use was associated with a significantly reduced body weight than other therapies (WMD: −3.47 kg, 95% CI: −3.96 to −2.98, *P* < 0.001). Similarly, both body mass index (BMI) (WMD: −1.25 kg/m^2^, 95% CI: −1.45 to −1.04, *P* < 0.001) and waist circumference (WMD: −2.59 cm, 95% CI: −3.09 to −2.08, *P* < 0.001) showed a significant reduction in semaglutide when compared to other therapies. No significant heterogeneity was observed within included studies (*I*^2^ = 17.6% for body weight, *I*^2^ = 30.5% for BMI, and *I*^2^ = 30.7% for waist circumference).

#### Blood pressure and pulse rate

For blood pressure control, systolic blood pressure (SBP), but not diastolic blood pressure (DBP) (WMD: −0.29 mmHg, 95% CI: −0.65 to 0.07, *P* = 0.113), revealed a significant reduction with semaglutide than other therapies (WMD: −2.55 mmHg, 95% CI: −3.22 to −1.88, *P* < 0.001). Unlike blood pressure, the use of semaglutide showed a significantly elevated pulse rate as compared with other therapies (WMD: 2.21 bpm, 95% CI: 1.54 to 2.88, *P* < 0.001), with moderate heterogeneity within studies (*I*^2^ = 67.6).

### Safety analysis

The following AEs were included for comparative analysis of safety and tolerability: AEs with varying degrees and AEs occurring in ≥ 5% patients based on predetermined terms or clinical significance. Finally, 22 AEs were selected, and results were presented in Table 2 and Table S4. The most significant results of the data from analyses were discussed next.

**Table 2:** Relative risk of adverse events reported for semaglutide in comparison with control.

	**No. of studies**	**Semaglutide therapy**	**Control therapy**	**Total**	**RR (SEM/CON)**	**95% CI (*P* value)**	**Homogeneity**	
							***I^2^* (%)**	***P***
**ALL ADVERSE EVENTS**								
Adverse events	9	4199/5774 (72.7%)	3027/3999 (75.6%)	7226/9773 (73.9%)	1.04	0.99 to 1.09 (0.002)	66.5	0.147
Serious adverse events	9	815/5774 (14.1%)	768/3999 (19.2%)	1583/9773 (16.1%)	0.93	0.86 to 1.01 (0.54)	0	0.084
Fatal adverse events	6	45/3458 (1.3%)	26/1812 (1.4%)	71/5270 (1.3%)	0.90	0.56 to 1.47 (0.52)	0	0.678
Moderate adverse events	5	595/2858 (20.8%)	269/1214 (22.1%)	864/4072 (21.2%)	1.01	0.90 to 1.15 (0.97)	0	0.833
Mild adverse events	5	1667/2858 (58.3%)	658/1214 (54.2%)	2325/4072 (57.0%)	1.08	0.96 to 1.22 (0.005)	73.1	0.194
AEs leading to discontinuation	9	507/5774 (8.7%)	199/3999 (4.9%)	706/9773 (7.2%)	2.07	1.58 to 2.73 (0.10)	39.5	<0.001
**ADVERSE EVENTS OCCURRING IN ≥ 5% PATIENTS BY PREFERRED TERM OR OTHER OF CLINICAL INTEREST**								
Gastrointestinal adverse events	7	2193/5107 (42.9%)	1026/3461 (29.6%)	3219/8568 (37.5%)	1.98	1.49 to 2.62 (<0.001)	92.3	<0.001
Nausea	8	1002/5511 (18.1%)	330/3866 (8.5%)	1332/9377 (14.2%)	2.56	1.76 to 3.74 (<0.001)	84	<0.001
Diarrhoea	8	772/5511 (14.0%)	353/3866 (9.1%)	1125/9377 (11.9%)	1.84	1.37 to 2.47 (0.001)	71.1	<0.001
Constipation	6	218/3141 (6.9%)	64/1857 (3.4%)	282/4998 (5.6%)	2.07	1.28 to 3.35 (0.05)	54.4	0.003
Abdominal discomfort	2	41/685 (5.9%)	0/343 (0%)	41/1028 (3.9%)	18.94	2.60 to 137.73 (0.62)	0	0.004
Decreased appetite	6	253/3230 (7.8%)	73/2113 (3.4%)	326/5343 (6.1%)	3.30	1.44 to 7.61 (<0.001)	80.4	0.005
Vomiting	7	476/5306 (8.9%)	169/3763 (4.4%)	645/9069 (7.1%)	2.16	1.54 to 3.03 (0.02)	60.4	<0.001
**Cardiovascular system**								
Cardiovascular*	5	338/3656 (9.2%)	377/2830 (13.3%)	715/6486 (11.0%)	0.88	0.78 to 1.01 (0.71)	0	0.066
Lipase increased	7	293/3863 (7.5%)	137/2217 (6.1%)	430/6080 (7.0%)	1.36	0.93 to 2.00 (0.01)	62.5	0.113
**Nervous system disorders**	2	54/927 (5.8%)	25/463 (5.3%)	79/1390 (5.6%)	1.08	0.68 to 1.71 (0.81)	0	0.75
Headache	5	166/2661 (6.2%)	98/1737 (5.6%)	264/4398 (6.0%)	1.23	0.91 to 1.65 (0.27)	22.4	0.176
**Other adverse events**								
Neoplasms	3	205/2946 (6.9%)	156/2176 (7.1%)	361/5122 (7.0%)	1.07	0.78 to1.46 (0.29)	18.6	0.673
Pancreatitis	5	15/4422 (0.3%)	12/2831 (0.4%)	27/7253 (0.3%)	0.82	0.36 to 1.88 (0.45)	0	0.641
Hypoglycaemia	5	411/3197 (12.8%)	367/2603 (14.0%)	778/5800 (13.4%)	1.07	0.94 to 1.21 (0.76)	0	0.317
Allergic reaction	2	114/2249 (5.0%)	124/2247 (5.5%)	238/4496 (5.2%)	0.92	0.72 to 1.78 (0.52)	0	0.51
Nasopharyngitis	7	424/3863 (10.9%)	239/2217 (10.7%)	663/6080 (10.9%)	0.86	0.74 to 0.99 (0.51)	0	0.04

Cardiovascular* means any events about cardiovascular or cardiac disorder, SEM, semaglutide; CON, control.

All included studies reported different degrees of AEs. The data showed that semaglutide did not increase the risk of any AEs (RR: 1.04, 95% CI: 0.99 to 1.09, *P* = 0.147), serious AEs (RR: 0.93, 95% CI: 0.86 to 1.01, *P* = 0.084), fatal AEs (RR: 0.90, 95% CI: 0.56 to 1.47, *P* = 0.678), moderate AEs (RR: 1.01, 95% CI: 0.90 to 1.15, *P* = 0.833), and mild AEs (RR: 1.08, 95% CI: 0.96 to 1.22, *P* = 0.194), with the exception of AEs leading to premature treatment discontinuation (RR: 2.07, 95% CI: 1.58 to 2.73, *P* < 0.001).

The incidence of gastrointestinal disorders was 42.9% (2193 of 5107) in the semaglutide group, while that was 29.6% (1026 of 3461) in the other therapies group. Thus, the presence of gastrointestinal disorders was considered as the most common AEs, and the pooled data showed a significantly higher risk with semaglutide than other therapies (RR = 1.98, 95% CI: 1.49 to 2.62, *P* < 0.001).

The total incidence of hypoglycemia was 12.8% (411 of 3,197) in patients treated with semaglutide, while that was 14.0% (367 of 2603) in other therapies group. In SUSTAIN 6 study, the incidence of hypoglycemia was 22.4% (369 of 1648) in semaglutide group and 21.2% (350/1649) in placebo group, respectively (Marso et al., 2016). Accordingly, the pooled data failed to show a significantly increased risk of hypoglycemia in patients taking semaglutide than those receiving other treatment (RR: 1.07, 95% CI: 0.94 to 1.21, *P* = 0.317).

Pancreatitis occurred with the incidence of 0.3% (15 of 4422) and 0.4% (12 of 2831) in semaglutide group and in other therapies group, respectively. No significantly higher risk was observed with semaglutide vs. other therapies (RR: 0.82, 95% CI: 0.36 to 1.88, *P* = 0.641).

The incidence of nasopharyngitis was slightly higher in semaglutide as compared to other therapies (10.9% vs.10.7%), with a corresponding RR of 0.86 (95%CI: 0.74 to 0.99, *P* = 0.04). No statistical difference was found in the incidence of other known AEs in terms of cardiovascular disorders (RR: 0.88, 95% CI: 0.78 to 1.01, *P* = 0.066), neoplasms (RR: 1.07, 95% CI: 0.78 to 1.46, *P* = 0.673), and nervous system disorders (RR: 1.08, 95% CI: 0.68 to 1.71, *P* = 0.75) between semaglutide and other therapies.

### Subgroup and sensitivity analysis

The results of semaglutide by different dosage (0.5 mg or 1.0 mg) and duration of follow up (< 30 weeks or more than 30 weeks) were consistent with the primacy analysis in terms of efficacy and safety (Tables S3, S4), some differences are discussed next.

Considering glycemic control by different treatment, the use of semaglutide significantly reduced HbA1c%, FPG, SMPG as well as PSMPG as compared to other therapies (placebo, sitagliptin, other GLP-1 RAs, and other oral anti-diabetic drug) except for FPG (WMD: −0.27 mmol/L, 95% CI: −0.94 to 0.41, *P* = 0.442) and SMPG (WMD: −0.31 mmol/L, 95% CI: −0.83 to 0.21, *P* = 0.249) with the comparison of insulin glargine. With regards to body weight control by different treatment, semaglutide was superior to all other therapies, including other once weekly GLP-1 RAs (body weight, WMD: −3.19 kg, 95% CI: 4.13 to 2.26, *P* < 0.001; BMI, WMD: −1.14 kg/m^2^, 95% CI: −1.47 to 0.81, *P* < 0.001; waist circumference, WMD: −2.33 cm, 95% CI: −2.86 to −1.81, *P* < 0.001). When regarding blood pressure control by different treatment, most of the results were in line with the primary analyses except for DBP as compared to sitagliptin (WMD: −0.86 mmHg, 95% CI: −1.60 to −0.13, *P* = 0.022) or other GLP-1 RAs (WMD: −1.04 mmHg, 95% CI: −2.05 to−0.03, *P* = 0.044).

Considering the risk of AEs by different treatment, most results were consistent with the primacy analyses, with the exception of a mildly increased risk of any AEs when compared with insulin glargine (RR: 1.10, 95% CI: 1.00 to 1.20, *P* = 0.040) or other oral antidiabetic drugs (RR: 1.22, 95% CI: 1.08 to 1.37, *P* = 0.001). Interestingly, semaglutide even slightly decreased the risk of serious AEs as compared to placebo (RR: 0.91, 95% CI: 0.83 to 0.99, *P* = 0.030). With respect to GI AEs by different treatment, no significantly increased risk was detected with semaglutide vs. sitagliptin (RR: 3.21, 95% CI: 0.86 to 11.97, *P* = 0.082) or other GLP-1 RAs (RR: 1.07, 95% CI: 0.94 to 1.23, *P* = 0.300).

The results of sensitivity analysis, as shown in Tables S5, S6, were not altered after excluding each of the studies or placebo-controlled studies.

### Publication bias

As shown in Figures S1A–C, visual inspection of funnel plots for the analyses showed a certain dissymmetry. Further quantitative analyses of Begg test and Egger test were performed to detect the publication bias at level of statistics. Finally, quantitative analyses failed to find the significant presence of publication bias except for HbA1c% (*P* = 0.029 for Egger test), PSMPG (*P* < 0.05 for both Begg test and Egger test), suggesting that publication bias was acceptable overall. However, the presence of sponsored bias was a concern because all included nine studies were sponsored by Novio Nodisk.

## Discussion

This study was a meta-analysis to comprehensively evaluate the efficacy and safety of once-weekly semaglutide, which included nine controlled phase III clinical studies with different comparators in patients with type 2 diabetes. Overall, the results of our study suggested that semaglutide had a preferable property of glycemic control, body weight control and blood pressure control compared with other therapies (placebo, sitagliptin, other GLP-1 RAs, insulin glargine, and other oral anti-diabetic drugs). Meanwhile, semaglutide did not increase different degrees of AEs, hypoglycemia, and pancreatitis, but induced a high risk of gastrointestinal AEs. The results were consistent across the key subgroups.

A meta-analysis on the focus of other GLP-1 RAs (dulaglutide, albiglutide, and released exenatide) was performed by Karagiannis T (Karagiannis and Liakos, 2015), which showed that other GLP-1 RAs can reduce HbA1c% by about 1% compared with placebo and 0.3–0.4% compared with other anti-diabetic drugs (Karagiannis and Liakos, 2015). Another meta-analysis of dulaglutide showed a significantly reduced HbA1c% level by 0.68% as compared to monotherapy (metformin and liraglutide) and by 0.51% as compared to add-on therapy (placebo, sitagliptin, exenatide, liraglutide, and glargine; Zhang et al., 2016). Our results revealed that semaglutide significantly reduced the value of HbA1c% by 0.93%, FPG by 1.15 mmol/L, SMPG by 1.19 mmol/L and PSMPG by 0.43 mmol/L when compared with other therapies. In a dose-finding study, semaglutide revealed a dose-dependent effects on the level of HbA1c% (Nauck et al., 2016). Of note, two up-to-date studies showed that semaglutide had a preferable property on glycemic control than other once-weekly GLP-1 RAs (released exenatide and dulaglutide) (Ahmann et al., 2018; Pratley et al., 2018). Semaglutide at the dosage of 1.0 mg can reduce mean HbA1c% by 1.5%, but released exenatide with the dosage of 2.0 mg can only reduce HbA1c% by 0.9%. Thus, the estimated treatment difference of semaglutide vs. released exenatide was −0.62% with HbA1c% and −0.84 mmol/L with FPG (Ahmann et al., 2018). Similarly, semaglutide was superior to dulaglutide by the reduction of HbA1c% about 0.40% regardless of low dosage or high dosage (Pratley et al., 2018). Several underline mechanisms might explain the reason of strongly hypoglycemic ability of semaglutide. Firstly, the short-acting GLP-1 RAs primarily lower postprandial plasma glucose by inhibiting gastric emptying, whereas long-acting GLP-1 RAs have a strong effects on FPG through mediating insulinotropic and glucagonostatic actions (Meier, 2012). In addition, semaglutide have the capacity to improve beta cell function and insulin sensitivity primarily via weight loss (Fonseca et al., 2017; Kapitza et al., 2017). A positive effect on insulin sensitivity and beta cell function might have also contributed to the improvement in glycaemic control with semaglutide vs. other anti-diabetic agents.

Cardiovascular diseases are the leading cause of premature mortality and disability accounting for nearly one third of all deaths worldwide with considerable impacts on body weight and hypertension (Sisti et al., 2017). As for body weight control, weight loss was observed across all the GLP-1 RAs. A network meta-analysis evaluating the ability of body weight reduction have demonstrated that the rank 1 was exenatide 10 μg twice daily (reduced 1.92 kg than placebo) and rank 2 was liraglutide 1.8 mg daily (reduced 0.98 kg than placebo) (Sun et al., 2015a). Furthermore, semaglutide lowered body weight more than liraglutide (−4.8 kg for semaglutide 1.6 mg/day vs. −2.6 kg for liraglutide 1.8 mg; Nauck et al., 2016). However, both albiglutide and dulaglutide had showed fewer efficacies than liraglutide in the matter of weight loss. For albiglutide, weight loss was 0.6 kg but 2.2 kg for liraglutide in a 26 week trial (Pratley et al., 2014). For dulaglutide, weight loss was 2.9 kg but 3.6 kg for liraglutide in another 26 weeks trial (Dungan et al., 2014). In this meta-analysis, semaglutide significantly reduced body weight of 3.47 kg when compared with other therapies. Further subgroup analysis also found that semaglutide lowered body weight much more efficacious than other GLP-1 RAs (−3.19 kg).

With respect to blood pressure control, the magnitude of SBP reduction was observed for all GLP-1 RAs. A previous study had shown that the reduction of nearly 5 mmHg of SBP was supposed to lower risk of major cardiovascular events and death (Patel et al., 2007). Another meta-analysis had demonstrated that all GLP-1 RAs could decrease SBP ranging from 1.84 mmHg to 4.60 mmHg, while only exenatide (10 μg twice daily) significantly reduced DBP by 1.08 mmHg (Sun et al., 2015c). In addition, both exenatide and liraglutide could increase heart rate by 2–3 beats/min (Sun et al., 2015c). In the present study, semaglutide could reduce SBP by 2.55 mmHg when compared with other therapies. Consistent with other GLP-1 RAs, an increased pulse rate of 2.21beats/min was observed in the semaglutide treatment. In a cardiovascular outcomes trial, patients receiving semaglutide had a significant 26% decreased risk of death on cardiovascular causes, nonfatal myocardial infarction, or nonfatal stroke than those receiving placebo (Marso et al., 2016). The underlying mechanism on this association remains unclear. Diabetes itself is associated with an increased risk of cardiovascular disease, and well-controlled blood glucose with semaglutide therapy may contribute to low cardiovascular events. Regarding increased pulse rate, it is a class effect of GLP-1 RAs. The possible mechanism of increased heart rate of GLP-1 RAs is related to the activate effect on myocytes in sinoatrial node or the sympathetic nervous system (Lorenz et al., 2017). However, it is of note that the increased heart rate was not associated with increased cardiovascular risks in previous studies (Tan et al., 2017).

Consistent with other GLP-1 RAs, semaglutide did not increase any AEs, fatal, moderate, and mild AEs (Karagiannis and Liakos, 2015; Zhang et al., 2016). The most commonly reported AEs with semaglutide were gastrointestinal disorders, mainly manifested as nausea, vomiting and diarrhea, abdominal discomfort, and decreased appetite. Generally, the majority of gastrointestinal events were mild or moderate in severity. When compared with other GLP-1RAs, semaglutide did not increase gastrointestinal events (RR: 1.07, 95%CI: 0.94 to 1.23, *P* = 0.3). Furthermore, the risk of AEs leading to premature treatment discontinuation was much higher in semaglutide than other therapies (RR: 2.07, 95%CI: 1.58 to 2.73, *P* < 0.001), and the most reasons were still gastrointestinal events. A previous meta-analysis had revealed that all GLP-1 RAs dose regimens significantly increased the incidence of gastrointestinal events (Sun et al., 2015b). Indeed, gastrointestinal effects are a class effect of GLP-1 RAs, and most patients can tolerate. The proportion of patients withdrawing from study due to treatment-emergent AEs (TEAEs) was increased with the incremental dosage of semaglutide (Sun et al., 2015b; Nauck et al., 2016). Thus, for those who did not tolerate semaglutide, a low dose initiation may be an optional choice. The possible mechanism of gastrointestinal events of GLP-1 RAs are as follows: (1) there is a strong relationship in the GLP-1 RAs class that short-acting GLP-1 RAs display a prominent ability to reduce gastric emptying, nevertheless long-acting GLP-1 RAs have better glycemic control ability and less effect on gastric emptying (Lau et al., 2015); (2) Enhanced GLP-1 concentration mediated the anorexigenic effect in the paraventricular hypothalamus (Liu et al., 2017).

Otherwise, hypoglycemia is a serious challenge and obstacle in the T2DM treatment. In this meta-analysis, semaglutide did not increase the risk of hypoglycemia as compared to other therapies (RR: 1.07, 95% CI: 0.94 to 1.21, *P* = 0.317), which was consistent with the findings in a recently published meta-analysis (Zhang et al., 2016).

There has been remaining controversial on the risk of pancreatitis caused by incretin-based drugs (Butler et al., 2013). Some early studies did not support an increased risk of pancreatitis in incretin-treated patients with T2DM, while other studies did agree that incretin-based therapies may associate with pancreatitis (Li et al., 2014; Monami et al., 2014; Giorda et al., 2015; Roshanov and Dennis, 2015). In this meta-analysis, no significantly higher risk was observed with semaglutide vs. other therapies (0.3% vs. 0.4%; RR: 0.82, 95% CI: 0.36 to 1.88, *P* = 0.641).

Interestingly, this study demonstrated that semaglutide slightly decreased the risk of nasopharyngitis (RR: 0.86, 95%CI: 0.74 to 0.99, *P* = 0.04). This is a controversial conclusion at present, and the mechanism is still unclear. The possible reason that GLP-1 can inhibit infiltration and inflammation in adipose tissue macrophage may explain this finding partly (Lee et al., 2012).

Accordingly, semaglutide might become an alternative in T2DM patients under several clinical scenarios, such as patients who are intolerant to metformin or other hypoglycemic agent, patients with overweight or hypertension, patients who exist obvious insulin resistance, and patients with cardiovascular high-risk factors.

This study had several limitations. Firstly, there were only 9 RCTs included in this meta-analysis, of which SUSTAIN 5 were only abstract that some data cannot be extracted. We also have not get access to the compliance data due to the exclusion of real-world studies, making powerful subgroup analysis unavailable. Secondly, several outcomes have heterogeneity in spite of the performance of subgroup and sensitivity analysis. Thirdly, the baseline characteristics of included studies were not the same, including background treatment, controls, dosage, and duration of follow up. Whereas, we have performed the corresponding subgroup analysis to assess potential effect modifiers in baseline characteristics, and the results failed to identify these potential confounding on the outcomes. Undeniably, residual confounding effects between included studies cannot be excluded absolutely. Otherwise, the duration of included studies was different, which may lead to certain bias. Finally, it must be admitted that these studies of semaglutide are all sponsored by Novio Nodisk, thus sponsorship bias may present in this study.

## Conclusion

Our meta-analysis illustrated that semaglutide could improve the control of blood glucose, body weight and blood pressure and did not increase the risk of hypoglycemia and pancreatitis. Overall, semaglutide was effective and acceptable in patients with T2DM except for a high risk of gastrointestinal disorders. The capacity of glycaemic control and body weight control of semaglutide appeared more effective than other GLP-1 receptor agonists. However, considering the number of included studies and potential limitations, more large-scale, well-designed RCT, real-world studies as well as HRQOL studies are needed to prove our findings.

## Author contributions

F-HS and HL exacted and analyzed the data and wrote the first draft of the protocol. MC helped with the design of the protocol. Z-LZ submitted the registration on PROSPERO. Z-CG revised the manuscript. X-YL is the guarantors for the publication and take the responsibility for the paper. All authors participated in read, and approved the final manuscript.

### Conflict of interest statement

The authors declare that the research was conducted in the absence of any commercial or financial relationships that could be construed as a potential conflict of interest.
