# Supplementary material for: Efficacy and Safety of Once-Weekly Semaglutide for the Treatment of Type 2 Diabetes: A Systematic Review and Meta-Analysis of Randomized Controlled Trials
Source: Front Pharmacol. 2018 Jun 4;9:576. doi: 10.3389/fphar.2018.00576 (PMC5994433; doi:10.3389/fphar.2018.00576)
Supplement: Supplementary file 1 [file Table_1.DOCX]

**TABLE S1.** Randomized controlled trials excluded from the meta-analysis with reasons for exclusion

| **NCT number(Study)** | **Drugs** | **Dosage form** | **Reason for exclusion** |
| --- | --- | --- | --- |
| NCT02212067(Kapitza C 2017)^1^ | Semaglutide | IV/SC | Phase I study and no adverse events data |
| NCT00833716(Nauck, M.A 2017)^2^ | Semaglutide | IV/SC | Phase I study and trial duration <24weeks |
| NCT02022254(Hausner H 2017)^3^ | Semaglutide | IV/SC | Phase I study and trial duration <24weeks |
| NCT02060266(Jensen L 2017)^4^ | Semaglutide | IV/SC | Phase I study and trial duration <24weeks |
| NCT01324505(Kapitza C 2014)^5^ | Semaglutide | IV/SC | Phase I study and trial duration <24weeks |
| NCT02212067(Kapitza, C 2017)^6^ | Semaglutide | IV/SC | Not phase III study and no adverse events data |
| NCT02079870(Blundell J2017)^7^ | Semaglutide | IV/SC | Obesity patients and phase I study |
| NCT 01923181(Davies M 2017)^8^ | Semaglutide | PO | Phase II study and oral medication |
| NCT00696657(Nauck MA 2016)^9^ | Semaglutide | IV/SC | Phase II study and trial duration <24weeks |
| NCT02461589 | Semaglutide | IV/SC | Phase II study and dose finding study |

*PO: oral; IV/SC: intravenous or subcutaneous*
