# Supplementary material for: Efficacy and Safety of Once-Weekly Semaglutide for the Treatment of Type 2 Diabetes: A Systematic Review and Meta-Analysis of Randomized Controlled Trials
Source: Front Pharmacol. 2018 Jun 4;9:576. doi: 10.3389/fphar.2018.00576 (PMC5994433; doi:10.3389/fphar.2018.00576)
Supplement: Supplementary file 2 [file Table_2.DOCX]

**TABLE S2** Quality assessment results of included randomized controlled trials.

| **Study** | **Random sequence generation** | **Allocation concealment** | **Blinding of participants and personnel** | **Blinding of outcome assessment** | **Incomplete outcome data** | **Selective reporting** | **Other bias** |
| --- | --- | --- | --- | --- | --- | --- | --- |
| Sorli C (SUSTAIN1)2017 | L | L | L | L | L | L | L |
| Ahrén B(SUSTAIN2)2017 | L | L | L | L | L | L | L |
| Ahmann AJ(SUSTAIN3)2018 | L | L | H | L | L | L | L |
| Aroda VR(SUSTAIN4)2017 | L | L | H | L | L | L | L |
| Marso SP(SUSTAIN6)2016 | L | L | L | L | L | L | L |
| Pratley(SUSTAIN7)2018 | L | L | H | L | L | L | L |
| Seino Y (SUSTAIN^TM^) 2017 | L | L | H | L | L | L | L |
| Kaku K (SUSTAIN^TM^)2018 | L | L | H | L | L | L | L |

*L: low risk; U: unclear risk; H: high risk; SUSTAIN5 only abstracts was available, thus we did not do quality assessment of this study.*
