# Supplementary material for: Efficacy and Safety of Once-Weekly Semaglutide for the Treatment of Type 2 Diabetes: A Systematic Review and Meta-Analysis of Randomized Controlled Trials
Source: Front Pharmacol. 2018 Jun 4;9:576. doi: 10.3389/fphar.2018.00576 (PMC5994433; doi:10.3389/fphar.2018.00576)
Supplement: Supplementary file 3 [file Table_3.DOCX]

**TABLE S3** Subgroup analysis of main efficacy outcomes of Semaglutide Versus Control

|  | subgroup | studies | WMD | 95% CI | *P* |
| --- | --- | --- | --- | --- | --- |
| Different controls |  |  |  |  |  |
| HbA1c% | placebo  sitagliptin  other GLP-1  insulin glargine  OAD | 2  2  2  1  1 | -1.09  -1.10  -0.47  -0.60  -1.23 | -1.48 to -0.69  -1.36 to -0.83  -0.60 to -0.34  -1.02 to -0.17  -1.51 to -0.94 | <0.001  <0.001  <0.001  0.006  <0.001 |
| FPG | placebo  sitagliptin  other GLP-1  insulin glargine  OAD | 2  1  2  1  1 | -1.71  -1.23  -0.57  -0.27  -1.84 | -2.06 to -1.35  -1.74 to -0.72  -0.86 to -0.27  -0.94 to 0.41  -2.21 to -1.48 | <0.001  <0.001  <0.001  0.442  <0.001 |
| SMPG | placebo  sitagliptin  other GLP-1  insulin glargine  OAD | 2  1  2  1  1 | -1.91  -1.15  -0.59  -0.31  -1.82 | -2.18 to -1.65  -1.59 to -1.07  -0.76 to -0.43  -0.83 to 0.21  -2.24 to -1.40 | <0.001  <0.001  <0.001  0.249  <0.001 |
| PSMPG | placebo  sitagliptin  other GLP-1  insulin glargine  OAD | 2  1  2  1  1 | -0.70  -0.28  -0.29  -0.52  -0.56 | -0.94 to -0.47  -0.48 to -0.08  -0.41 to -0.16  -0.78 to -0.27  -0.87 to -0.25 | <0.001  0.005  <0.001  <0.001  <0.001 |
| Body weight | placebo  sitagliptin  other GLP-1  insulin glargine  OAD | 2  2  2  1  1 | -3.42  -3.16  -3.19  -5.47  -2.71 | -4.29 to -2.54  -4.18 to -2.15  -4.13 to -2.26  -7.15 to -3.80  -4.43 to -1.00 | <0.001  <0.001  <0.001  <0.001  0.002 |
| BMI | placebo  sitagliptin  other GLP-1  insulin glargine  OAD | 1  2  2  1  1 | -1.11  -1.19  -1.14  -1.97  -1.03 | -1.40 to -0.81  -1.56 to -0.82  -1.47 to -0.81  -2.56 to -1.37  -1.69 to -0.38 | <0.001  <0.001  <0.001  <0.001  0.002 |
| Waist circumference | placebo  sitagliptin  other GLP-1  insulin glargine  OAD | 1  2  2  1  1 | -2.01  -2.97  -2.33  -4.09  -2.40 | -3.11 to -0.91  -3.84 to -2.11  -2.86 to -1.81  -5.40 to -2.78  -3.46 to-1.34 | <0.001  <0.001  <0.001  <0.001  <0.001 |
| DBP | placebo  sitagliptin  other GLP-1  insulin glargine  OAD | 2  2  2  1  1 | -0.02  -0.86  -1.04  0.25  -0.33 | -0.59 to 0.54  -1.60 to -0.13  -2.05 to -0.03  -0.59 to 1.09  -1.52 to 0.86 | 0.939  0.022  0.044  0.553  0.589 |
| SBP | placebo  sitagliptin  other GLP-1  insulin glargine  OAD | 3  2  2  1  1 | -2.25  -3.38  -1.60  -3.23  -2.99 | -3.52 to -0.99  -4.57 to -2.18  -2.86 to -0.33  -4.61 to -1.85  -4.90 to -1.07 | <0.001  <0.001  0.013  <0.001  0.002 |
| Pulse rate | placebo  sitagliptin  other GLP-1  insulin glargine  OAD | 3  2  2  1  1 | 2.50  2.39  1.03  2.77  2.97 | 1.86 to 3.14  0.81 to 3.97  0.27 to 1.80  1.85 to 3.69  1.63 to 4.30 | <0.001  0.003  0.008  <0.001  <0.001 |
| Different dosage |  | study | WMD | 95%CI | p |
| HbA1c% | Sem0.5mg  Sem1.0mg | 7  8 | -0.75  -1.03 | -0.99 to -0.51  -1.29 to -0.77 | <0.001  <0.001 |
| FPG | Sem0.5mg  Sem1.0mg | 6  7 | -0.98  -1.30 | -1.61 to -0.36  -1.54 to -0.77 | 0.002  <0.001 |
| SMPG | Sem0.5mg  Sem1.0mg | 6  7 | -1.05  -1.33 | -1.60 to -0.51  -1.80 to -0.86 | <0.001  <0.001 |
| PSMPG | Sem0.5mg  Sem1.0mg | 6  7 | -0.33  -0.51 | -0.45 to -0.21  -0.70 to -0.33 | <0.001  <0.001 |
| Body weight | Sem0.5mg  Sem1.0mg | 7  8 | -2.72  -4.18 | -3.46 to -1.97  -4.89 to -3.48 | <0.001  <0.001 |
| BMI | Sem0.5mg  Sem1.0mg | 6  7 | -0.99  -1.51 | -1.31 to -0.66  -1.81 to -1.21 | <0.001  <0.001 |
| Waist circumference | Sem0.5mg  Sem1.0mg | 6  7 | -2.31  -3.26 | -2.85 to -1.78  -3.96 to -2.56 | <0.001  <0.001 |
| DBP | Sem0.5mg  Sem1.0mg | 7  8 | -0.23  -0.61 | -0.72 to 0.26  -1.24 to 0.02 | 0.365  0.059 |
| SBP | Sem0.5mg  Sem1.0mg | 8  9 | -1.90  -3.13 | -2.68 to -1.13  -3.97 to -2.29 | <0.001  <0.001 |
| Pulse rate | Sem0.5mg  Sem1.0mg | 8  9 | 1.86  2.59 | 1.24 to2.49  1.77 to 3.40 | <0.001  <0.001 |
| Different follow up |  |  |  |  |  |
| HbA1c% | Less than 30w  More than 30w | 3  5 | -1.07  -0.82 | -1.48 to -0.67  -1.04 to -0.61 | <0.001  <0.001 |
| FPG | Less than 30w  More than 30w | 3  4 | -1.19  -1.13 | -1.91 to -0.48  -1.59 to -0.67 | 0.001  <0.001 |
| SMPG | Less than 30w  More than 30w | 3  4 | -1.36  -1.09 | -2.10 to -0.62  -1.46 to -0.71 | <0.001  <0.001 |
| PSMPG | Less than 30w  More than 30w | 3  4 | -0.61  -0.31 | -0.78 to -0.44  -0.40 to -0.22 | <0.001  <0.001 |
| Body weight | Less than 30w  More than 30w | 3  5 | -3.92  -3.21 | -5.21 to -2.63  -3.80 to -2.62 | <0.001  <0.001 |
| BMI | Less than 30w  More than 30w | 3  4 | -1.42  -1.14 | -1.87 to -0.96  -1.39 to -0.89 | <0.001  <0.001 |
| Waist circumference | Less than 30w  More than 30w | 3  4 | -3.18  -2.52 | -4.07 to -2.28  -3.03 to -2.01 | <0.001  <0.001 |
| DBP | Less than 30w  More than 30w | 3  5 | -0.14  -0.50 | -0.79 to 0.50  -0.95 to -0.06 | 0.664  0.027 |
| SBP | Less than 30w  More than 30w | 4  5 | -3.25  -2.23 | -4.43 to -2.07  -2.88 to -1.58 | <0.001  <0.001 |
| Pulse rate | Less than 30w  More than 30w | 4  5 | 3.12  1.68 | 2.43 to 3.80  1.15 to 2.20 | <0.001  <0.001 |
