# Supplementary material for: Efficacy and Safety of Once-Weekly Semaglutide for the Treatment of Type 2 Diabetes: A Systematic Review and Meta-Analysis of Randomized Controlled Trials
Source: Front Pharmacol. 2018 Jun 4;9:576. doi: 10.3389/fphar.2018.00576 (PMC5994433; doi:10.3389/fphar.2018.00576)
Supplement: Supplementary file 4 [file Table_4.DOCX]

**TABLE S4.** Subgroup analysis of main safety outcomes of Semaglutide Versus Control

|  | subgroup | studies | WMD | 95%CI | *P* |
| --- | --- | --- | --- | --- | --- |
| Different controls |  |  |  |  |  |
| Adverse events | placebo  sitagliptin  other GLP-1  insulin glargine  OAD | 3  2  2  1  1 | 0.99  1.03  0.99  1.10  1.22 | 0.86 to 1.14  0.97 to 1.11  0.94 to 1.05  1.00 to 1.20  1.08 to 1.37 | 0.885  0.331  0.829  0.040  0.001 |
| Serious adverse events | placebo  sitagliptin  other GLP-1  insulin glargine  OAD | 3  2  2  1  1 | 0.91  1.08  1.15  1.08  0.97 | 0.83 to 0.99  0.72 to 1.63  0.64 to 1.09  0.63 to 1.86  0.46 to 2.05 | 0.030  0.713  0.638  0.781  0.934 |
| Fatal adverse events | sitagliptin  other GLP-1  insulin glargine  OAD | 2  1  1  1 | 0.50  0.50  1.08  0.90 | 0.10 to 2.45  0.09 to 2.71  0.63 to 1.86  0.56 to 1.47 | 0.391  0.419  0.781  0.326 |
| Moderate adverse events | placebo  sitagliptin  insulin glargine  OAD | 1  2  1  1 | 0.90  1.01  1.05  0.97 | 0.59 to 1.38  0.84 to 1.22  0.86 to 1.27  0.57 to 1.64 | 0.623  0.878  0.659  0.900 |
| Mild adverse events | placebo  sitagliptin  insulin glargine  OAD | 1  2  1  1 | 0.80  1.03  1.17  1.28 | 0.61 to 1.05  0.95 to 1.11  1.04 to 1.30  1.13 to 1.47 | 0.102  0.491  0.007  <0.001 |
| GI adverse events | placebo  sitagliptin  other GLP-1  insulin glargine  OAD | 2  2  1  1  1 | 1.47  3.21  1.07  2.82  2.70 | 1.35 to 1.59  0.86 to 11.97  0.94 to 1.23  2.17 to 3.65  1.87 to 3.90 | <0.001  0.082  0.300  <0.001  <0.001 |
| AEs leading to discontinuation | placebo  sitagliptin  other GLP-1  insulin glargine  OAD | 3  2  2  1  1 | 1.97  3.06  1.44  5.86  2.50 | 1.59 to 2.44  1.76 to 5.32  1.06 to 1.96  2.13 to 16.13  0.91 to 6.85 | <0.001  <0.001  0.021  0.001  0.075 |
| Different dosage |  |  |  |  |  |
| Adverse events | Sem0.5mg  Sem1.0mg | 8  9 | 1.06  1.03 | 0.99 to 1.14  0.98 to 1.09 | 0.077  0.296 |
| Serious adverse events | Sem0.5mg  Sem1.0mg | 8  9 | 0.93  0.93 | 0.84 to 1.03  0.86 to 1.00 | 0.186  0.139 |
| Fatal adverse events | Sem0.5mg  Sem1.0mg | 6  6 | 1.06  0.80 | 0.61 to 1.83  0.44 to 1.44 | 0.838  0.451 |
| Moderate adverse events | Sem0.5mg  Sem1.0mg | 5  5 | 1.03  1.00 | 0.90 to 1.19  0.86 to 1.15 | 0.675  0.965 |
| Mild adverse events | Sem0.5mg  Sem1.0mg | 5  5 | 1.08  1.10 | 0.96 to 1.22  0.97 to 1.24 | 0.219  0.134 |
| GI adverse events | Sem0.5mg  Sem1.0mg | 7  7 | 1.98  2.02 | 1.52 to 2.58  1.45 to 2.81 | <0.001  <0.001 |
| AEs leading to discontinuation | Sem0.5mg  Sem1.0mg | 8  9 | 1.91  2.37 | 1.54 to 2.36  1.68 to 3.35 | <0.001  <0.001 |
| Different follow up |  |  |  |  |  |
| Adverse events | Less than 30w  More than 30w | 4  5 | 1.06  1.02 | 0.94 to 1.19  0.97 to 1.08 | 0.383  0.389 |
| Serious adverse events | Less than 30w  More than 30w | 4  5 | 1.12  0.97 | 0.76 to 1.66  0.82 to 1.15 | 0.564  0.719 |
| Fatal adverse events | Less than 30w  More than 30w | 3  3 | 1.08  0.45 | 0.63 to 1.86  0.15 to 1.31 | 0.781  0.143 |
| Moderate adverse events | Less than 30w  More than 30w | 3  2 | 1.02  1.00 | 0.86 to 1.22  0.84 to 1.20 | 0.797  0.970 |
| Mild adverse events | Less than 30w  More than 30w | 3  2 | 1.03  1.14 | 0.86 to 1.24  0.91 to 1.43 | 0.759  0.260 |
| GI adverse events | Less than 30w  More than 30w | 3  4 | 2.95  1.58 | 1.74 to 4.99  1.21 to 2.06 | <0.001  0.001 |
| AEs leading to discontinuation | Less than 30w  More than 30w | 4  5 | 3.86  1.84 | 2.07 to 7.40  1.45 to 2.34 | <0.001  <0.001 |
