# Supplementary material for: Efficacy and Safety of Once-Weekly Semaglutide for the Treatment of Type 2 Diabetes: A Systematic Review and Meta-Analysis of Randomized Controlled Trials
Source: Front Pharmacol. 2018 Jun 4;9:576. doi: 10.3389/fphar.2018.00576 (PMC5994433; doi:10.3389/fphar.2018.00576)
Supplement: Supplementary file 5 [file Table_5.DOCX]

**TABLE S5.** Sensitivity analysis for main efficacy outcomes

| **Study omitted** | **RR** | **95%CI** |
| --- | --- | --- |
| **HbA1c%** | RR | 95%CI |
| Sorli C et al.,2017 (SUSTAIN1) | -0.84 | -1.11 to -0.56 |
| Ahrén B et al., 2017(SUSTAIN2) | -0.93 | -1.28 to -0.58 |
| Ahmann AJ et al.,2018 (SUSTAIN3) | -0.98 | -1.36 to -0.59 |
| Aroda VR et al.,2017(SUSTAIN4) | -0.97 | -1.31 to -0.63 |
| Marso SP et al.,2016(SUSTAIN6) | -0.94 | -1.28 to -0.60 |
| Pratley et al.,2018 (SUSTAIN7) | -1.01 | -1.30 to -0.73 |
| Seino Y et al.,2018 (SUSTAIN^TM^) | -0.88 | -1.20 to -0.56 |
| Kaku K et al.2018 (SUSTAIN^TM^) | -0.88 | -1.21 to -0.56 |
| Placebo-controlled studies | -0.83 | -1.14 to -0.53 |
| **FPG** |  |  |
| Sorli C et al.,2017 (SUSTAIN1) | -1.02 | -1.58 to -0.49 |
| Ahrén B et al., 2017(SUSTAIN2) | -1.13 | -1.73 to -0.54 |
| Ahmann AJ et al.,2018 (SUSTAIN3) | -1.20 | -1.83 to -0.58 |
| Aroda VR et al.,2017(SUSTAIN4) | -1.28 | -1.83 to -0.72 |
| Rodbard H et al.,2017 (SUSTAIN 5) | -1.10 | -1.67 to -0.53 |
| Pratley et al.,2018 (SUSTAIN7) | -1.29 | -1.77 to -0.81 |
| Kaku K et al.2018 (SUSTAIN^TM^) | -1.03 | -1.56 to -0.49 |
| Placebo-controlled studies | -0.94 | -1.52 to -0.36 |
| **SMPG mean** |  |  |
| Sorli C et al.,2017 (SUSTAIN1) | -1.08 | -1.56 to -0.60 |
| Ahrén B et al., 2017(SUSTAIN2) | -1.20 | -1.77 to -0.63 |
| Ahmann AJ et al.,2018 (SUSTAIN3) | -1.27 | -1.86 to -0.68 |
| Aroda VR et al.,2017(SUSTAIN4) | -1.33 | -1.85 to -0.80 |
| Rodbard H et al.,2017 (SUSTAIN 5) | -1.06 | -1.55 to -0.57 |
| Pratley et al.,2018 (SUSTAIN7) | -1.31 | -1.82 to -0.80 |
| Kaku K et al.2018 (SUSTAIN^TM^) | -1.09 | -1.59 to -0.58 |
| Placebo-controlled studies | -0.91 | -1.35 to -0.46 |
| **SMPG (Postprandial increments)** |  |  |
| Sorli C et al.,2017 (SUSTAIN1) | -0.42 | -0.56 to -0.27 |
| Ahrén B et al., 2017(SUSTAIN2) | -0.47 | -0.63 to -0.31 |
| Ahmann AJ et al.,2018 (SUSTAIN3) | -0.47 | -0.63 to -0.32 |
| Aroda VR et al.,2017(SUSTAIN4) | -0.42 | -0.58 to -0.27 |
| Rodbard H et al.,2017 (SUSTAIN5) | -0.38 | -0.49 to -0.26 |
| Pratley et al.,2018 (SUSTAIN7) | -0.47 | -0.64 to -0.30 |
| Kaku K et al.2018 (SUSTAIN^TM^) | -0.42 | -0.57 to -0.27 |
| Placebo-controlled studies | -0.35 | -0.46 to -0.24 |
| **body weight** |  |  |
| Sorli C et al.,2017 (SUSTAIN1) | -3.56 | -4.15 to -2.96 |
| Ahrén B et al., 2017(SUSTAIN2) | -3.48 | -4.03 to -2.93 |
| Ahmann AJ et al.,2018 (SUSTAIN3) | -3.37 | -3.97 to -2.78 |
| Aroda VR et al.,2017(SUSTAIN4) | -3.33 | -3.77 to -2.88 |
| Marso SP et al.,2016(SUSTAIN6) | -3.45 | -4.02 to -2.89 |
| Pratley et al.,2018 (SUSTAIN7) | -3.55 | -4.09 to -3.01 |
| Seino Y et al.,2018 (SUSTAIN^TM^) | -3.51 | -4.05 to -2.96 |
| Kaku K et al.2018 (SUSTAIN^TM^) | -3.53 | -4.05 to -3.00 |
| Placebo-controlled studies | -3.54 | -4.25 to -2.83 |
| **BMI** |  |  |
| Sorli C et al.,2017 (SUSTAIN1) | -1.29 | -1.54 to -1.04 |
| Ahrén B et al., 2017(SUSTAIN2) | -1.25 | -1.48 to -1.02 |
| Ahmann AJ et al.,2018 (SUSTAIN3) | -1.22 | -1.47 to -0.96 |
| Aroda VR et al.,2017(SUSTAIN4) | -1.19 | -1.35 to -1.02 |
| Pratley et al.,2018 (SUSTAIN7) | -1.29 | -1.52 to -1.06 |
| Seino Y et al.,2018 (SUSTAIN^TM^) | -1.26 | -1.50 to -1.03 |
| Kaku K et al.2018 (SUSTAIN^TM^) | -1.27 | -1.50 to -1.05 |
| Placebo-controlled studies | -1.29 | -1.54 to -1.04 |
| **Waist circumference** |  |  |
| Sorli C et al.,2017 (SUSTAIN1) | -2.70 | -3.28 to -2.13 |
| Ahrén B et al., 2017(SUSTAIN2) | -2.57 | -3.15 to -2.00 |
| Ahmann AJ et al.,2018 (SUSTAIN3) | -2.58 | -3.21 to -1.95 |
| Aroda VR et al.,2017(SUSTAIN4) | -2.35 | -2.77 to -1.94 |
| Pratley et al.,2018 (SUSTAIN7) | -2.76 | -3.33 to -2.19 |
| Seino Y et al.,2018 (SUSTAIN^TM^) | -2.59 | -3.13 to -2.04 |
| Kaku K et al.2018 (SUSTAIN^TM^) | -2.65 | -3.27 to -2.04 |
| Placebo-controlled studies | -2.70 | -3.28 to -2.13 |
| **DBP** |  |  |
| Sorli C et al.,2017 (SUSTAIN1) | -0.30 | -0.71 to 0.11 |
| Ahrén B et al., 2017(SUSTAIN2) | -0.17 | -0.56 to 0.23 |
| Ahmann AJ et al.,2018 (SUSTAIN3) | -0.24 | -0.61 to 0.14 |
| Aroda VR et al.,2017(SUSTAIN4) | -0.41 | -0.80 to -0.01 |
| Marso SP et al.,2016(SUSTAIN6) | -0.48 | -0.91 to -0.04 |
| Pratley et al.,2018 (SUSTAIN7) | -0.26 | -0.62 to 0.11 |
| Seino Y et al.,2018 (SUSTAIN^TM^) | -0.29 | -0.68 to 0.10 |
| Kaku K et al.2018 (SUSTAIN^TM^) | -0.33 | -0.75 to 0.10 |
| Placebo-controlled studies | -0.47 | -0.93 to -0.01 |
| **SBP** |  |  |
| Sorli C et al.,2017 (SUSTAIN1) | -2.67 | -3.33 to -2.00 |
| Ahrén B et al., 2017(SUSTAIN2) | -2.45 | -3.23 to -1.67 |
| Ahmann AJ et al.,2018 (SUSTAIN3) | -2.58 | -3.34 to -1.81 |
| Aroda VR et al.,2017(SUSTAIN4) | -2.42 | -3.17 to -1.67 |
| Rodbard H et al.,2017 (SUSTAIN5) | -2.45 | -3.08 to -1.81 |
| Marso SP et al.,2016(SUSTAIN6) | -2.68 | -3.45 to -1.92 |
| Pratley et al.,2018 (SUSTAIN7) | -2.73 | -3.36 to -2.10 |
| Seino Y et al.,2018 (SUSTAIN^TM^) | -2.48 | -3.17 to -1.80 |
| Kaku K et al.2018 (SUSTAIN^TM^) | -2.50 | -3.26 to -1.75 |
| Placebo-controlled studies | -2.74 | -3.43 to -2.04 |
| **Pulse rate** |  |  |
| Sorli C et al.,2017 (SUSTAIN1) | 2.14 | 1.43 to 2.86 |
| Ahrén B et al., 2017(SUSTAIN2) | 2.39 | 1.69 to 3.08 |
| Ahmann AJ et al.,2018 (SUSTAIN3) | 2.36 | 1.66 to 3.07 |
| Aroda VR et al.,2017(SUSTAIN4) | 2.14 | 1.40 to 2.88 |
| Rodbard H et al.,2017 (SUSTAIN5) | 2.18 | 1.49 to 2.87 |
| Marso SP et al.,2016(SUSTAIN6) | 2.24 | 1.42 to 3.06 |
| Pratley et al.,2018 (SUSTAIN7) | 2.38 | 1.68 to 3.08 |
| Seino Y et al.,2018 (SUSTAIN^TM^) | 2.00 | 1.39 to 2.62 |
| Kaku K et al.2018 (SUSTAIN^TM^) | 2.13 | 1.41 to 2.85 |
| Placebo-controlled studies | 2.09 | 1.15 to 3.03 |
