# Supplementary material for: Efficacy and Safety of Once-Weekly Semaglutide for the Treatment of Type 2 Diabetes: A Systematic Review and Meta-Analysis of Randomized Controlled Trials
Source: Front Pharmacol. 2018 Jun 4;9:576. doi: 10.3389/fphar.2018.00576 (PMC5994433; doi:10.3389/fphar.2018.00576)
Supplement: Supplementary file 6 [file Table_6.DOCX]

**TABLE S6.** Sensitivity analysis for main safety outcomes

| **Study omitted** | **RR** | **95% CI** |
| --- | --- | --- |
| **Adverse events** | RR | 95% CI |
| Sorli C et al.,2017 (SUSTAIN1) | 1.05 | 1.00 to 1.10 |
| Ahrén B et al., 2017(SUSTAIN2) | 1.04 | 0.98 to 1.11 |
| Ahmann AJ et al.,2018 (SUSTAIN3) | 1.05 | 0.99 to 1.11 |
| Aroda VR et al.,2017(SUSTAIN4) | 1.03 | 0.98 to 1.08 |
| Rodbard H et al.,2017 (SUSTAIN 5) | 1.03 | 0.98 to 1.08 |
| Marso SP et al.,2016(SUSTAIN6) | 1.05 | 0.99 to 1.12 |
| Pratley et al.,2018 (SUSTAIN7) | 1.04 | 0.99 to 1.11 |
| Seino Y et al.,2018 (SUSTAIN^TM^) | 1.03 | 0.98 to 1.09 |
| Kaku K et al.2018 (SUSTAIN^TM^) | 1.02 | 0.98 to 1.06 |
| Placebo-controlled studies | 1.06 | 0.99 to 1.12 |
| **Serious adverse events** |  |  |
| Sorli C et al.,2017 (SUSTAIN1) | 0.93 | 0.86 to 1.01 |
| Ahrén B et al., 2017(SUSTAIN2) | 0.93 | 0.85 to 1.01 |
| Ahmann AJ et al.,2018 (SUSTAIN3) | 0.92 | 0.84 to 1.00 |
| Aroda VR et al.,2017(SUSTAIN4) | 0.93 | 0.85 to 1.01 |
| Rodbard H et al.,2017 (SUSTAIN 5) | 0.93 | 0.85 to 1.01 |
| Marso SP et al.,2016(SUSTAIN6) | 1.08 | 0.88 to 1.33 |
| Pratley et al.,2018 (SUSTAIN7) | 0.93 | 0.86 to 1.02 |
| Seino Y et al.,2018 (SUSTAIN^TM^) | 0.93 | 0.85 to 1.01 |
| Kaku K et al.2018 (SUSTAIN^TM^) | 0.93 | 0.85 to 1.01 |
| Placebo-controlled studies | 1.08 | 0.87 to 1.35 |
| **Fatal adverse events** |  |  |
| Sorli C et al.,2017 (SUSTAIN1) | 0.90 | 0.56 to 1.47 |
| Ahrén B et al., 2017(SUSTAIN2) | 0.96 | 0.58 to 1.60 |
| Aroda VR et al.,2017(SUSTAIN4) | 0.45 | 0.15 to 1.31 |
| Pratley et al.,2018 (SUSTAIN7) | 0.95 | 0.57 to 1.58 |
| Seino Y et al.,2018 (SUSTAIN^TM^) | 0.90 | 0.56 to 1.47 |
| Kaku K et al.2018 (SUSTAIN^TM^) | 0.94 | 0.57 to 1.54 |
| Placebo-controlled studies | 0.90 | 0.56 to1.47 |
| **Moderate adverse events** |  |  |
| Sorli C et al.,2017 (SUSTAIN1) | 1.03 | 0.90 to 1.17 |
| Ahrén B et al., 2017(SUSTAIN2) | 1.02 | 0.86 to 1.20 |
| Aroda VR et al.,2017(SUSTAIN4) | 0.99 | 0.85 to 1.17 |
| Seino Y et al.,2018 (SUSTAIN^TM^) | 1.01 | 0.89 to 1.15 |
| Kaku K et al.2018 (SUSTAIN^TM^) | 1.02 | 0.89 to 1.16 |
| Placebo-controlled studies | 1.03 | 0.90 to 1.17 |
| **Mild adverse events** |  |  |
| Sorli C et al.,2017 (SUSTAIN1) | 1.13 | 1.01 to 1.25 |
| Ahrén B et al., 2017(SUSTAIN2) | 1.10 | 0.94 to 1.28 |
| Aroda VR et al.,2017(SUSTAIN4) | 1.05 | 0.90 to 1.23 |
| Seino Y et al.,2018 (SUSTAIN^TM^) | 1.08 | 0.94 to 1.25 |
| Kaku K et al.2018 (SUSTAIN^TM^) | 1.04 | 0.93 to 1.16 |
| Placebo-controlled studies | 1.13 | 1.01 to 1.25 |
| **GI adverse events** |  |  |
| Sorli C et al.,2017 (SUSTAIN1) | 2.00 | 1.47 to 2.72 |
| Ahrén B et al., 2017(SUSTAIN2) | 2.05 | 1.46 to 2.88 |
| Aroda VR et al.,2017(SUSTAIN4) | 1.81 | 1.38 to 2.37 |
| Marso SP et al.,2016(SUSTAIN6) | 2.21 | 1.42 to 3.43 |
| Pratley et al.,2018 (SUSTAIN7) | 2.25 | 1.64 to 3.09 |
| Seino Y et al.,2018 (SUSTAIN^TM^) | 1.79 | 1.37 to 2.34 |
| Kaku K et al.2018 (SUSTAIN^TM^) | 1.87 | 1.40 to 2.51 |
| Placebo-controlled studies | 2.30 | 1.39 to 3.79 |
| **AEs leading to discontinuation** |  |  |
| Sorli C et al.,2017 (SUSTAIN1) | 2.12 | 1.57 to 2.85 |
| Ahrén B et al., 2017(SUSTAIN2) | 1.95 | 1.47 to 2.60 |
| Ahmann AJ et al.,2018 (SUSTAIN3) | 2.25 | 1.69 to 2.99 |
| Aroda VR et al.,2017(SUSTAIN4) | 1.89 | 1.53 to 2.34 |
| Rodbard H et al.,2017 (SUSTAIN5) | 2.02 | 1.54 to 2.64 |
| Marso SP et al.,2016(SUSTAIN6) | 2.26 | 1.52 to 3.37 |
| Pratley et al.,2018 (SUSTAIN7) | 2.27 | 1.63 to 3.15 |
| Seino Y et al.,2018 (SUSTAIN^TM^) | 2.05 | 1.54 to 2.72 |
| Kaku K et al.2018 (SUSTAIN^TM^) | 2.07 | 1.54 to 2.79 |
| Placebo-controlled studies | 2.23 | 1.44 to 3.46 |
