# Supplementary figures and images for: Efficacy and Safety of Once-Weekly Semaglutide for the Treatment of Type 2 Diabetes: A Systematic Review and Meta-Analysis of Randomized Controlled Trials
Source: Front Pharmacol. 2018 Jun 4;9:576. doi: 10.3389/fphar.2018.00576 (PMC5994433; doi:10.3389/fphar.2018.00576)

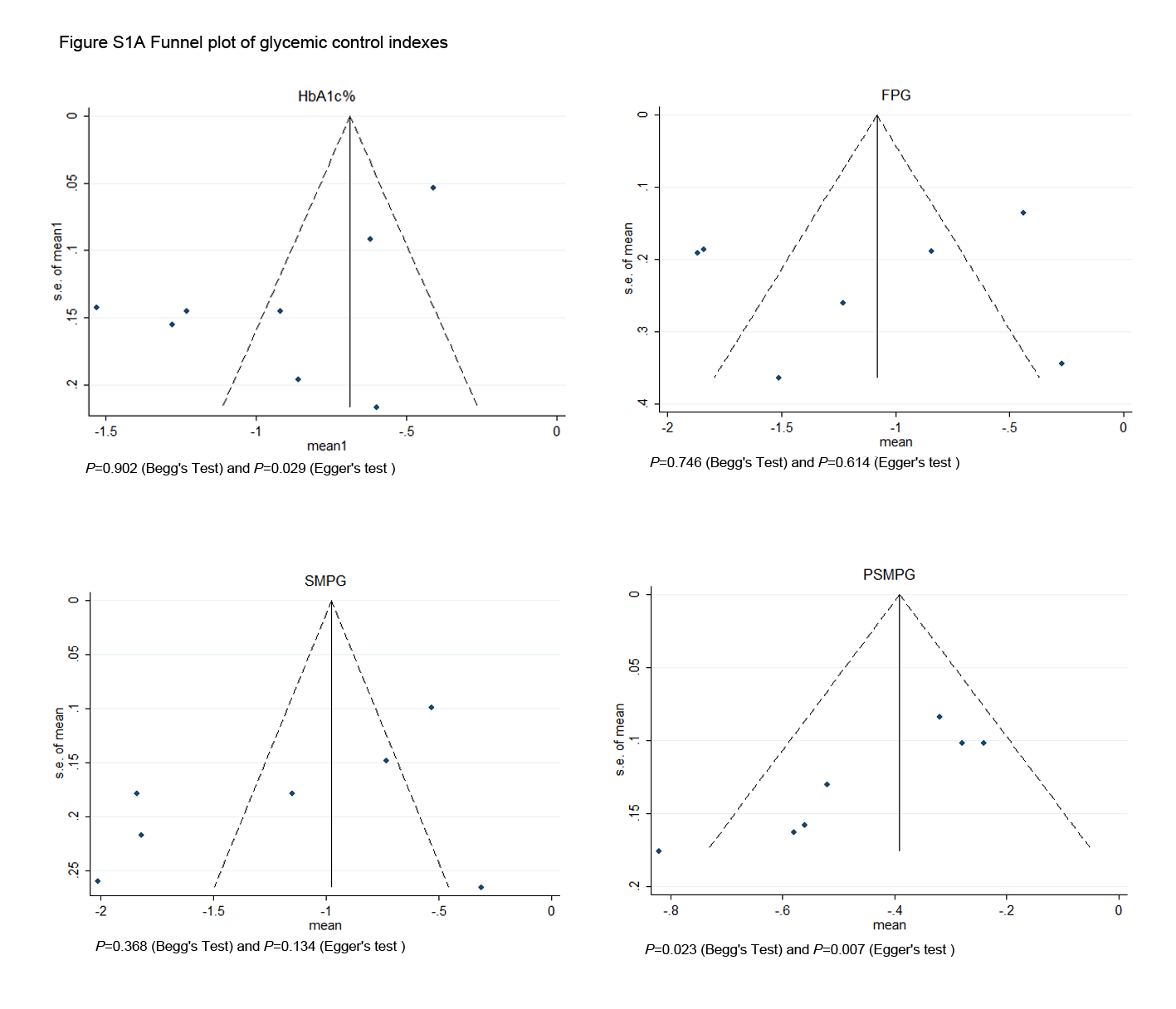

Supplement: Supplementary file 7 [file Image_1.tif]

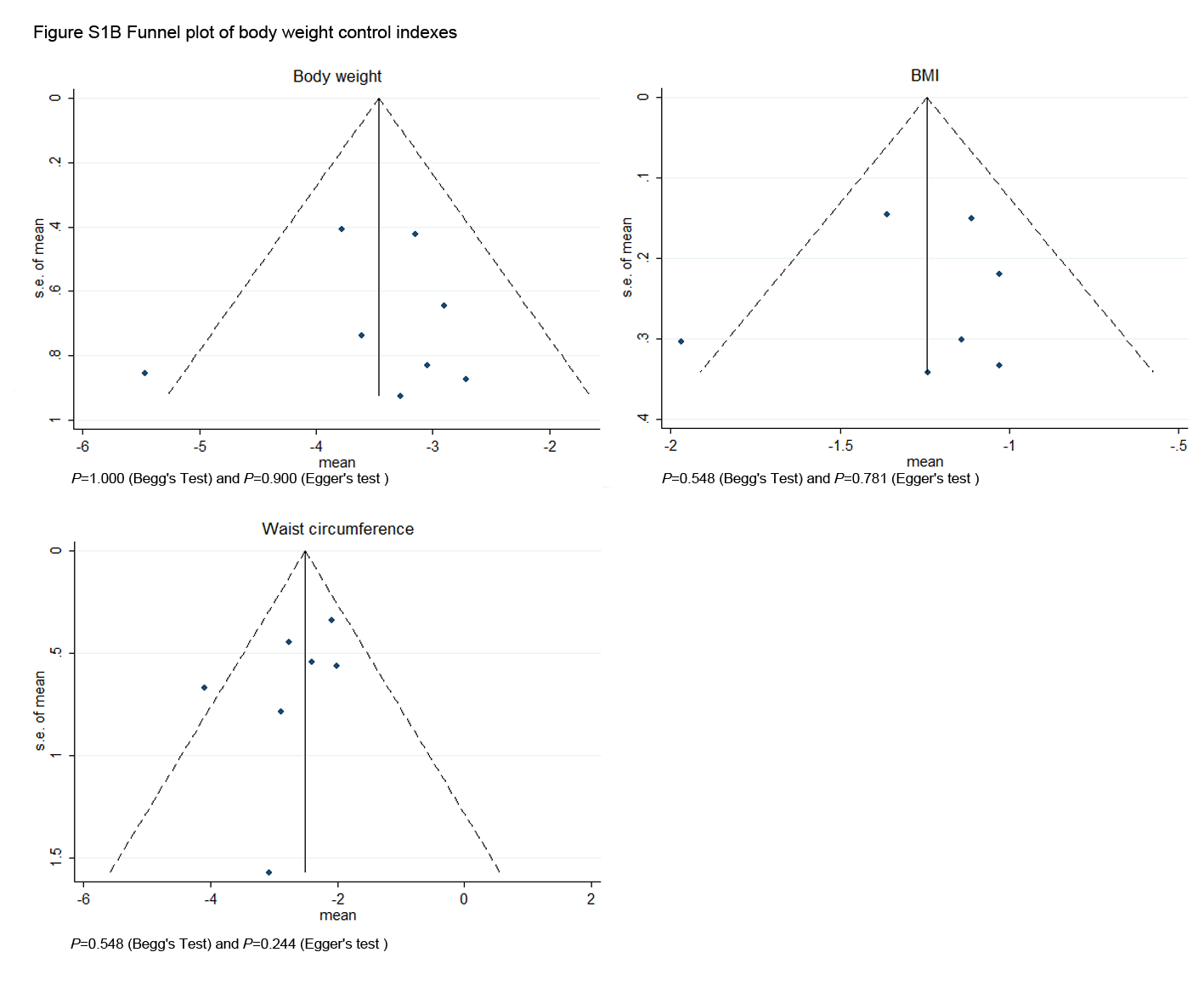

Supplement: Supplementary file 8 [file Image_2.tif]

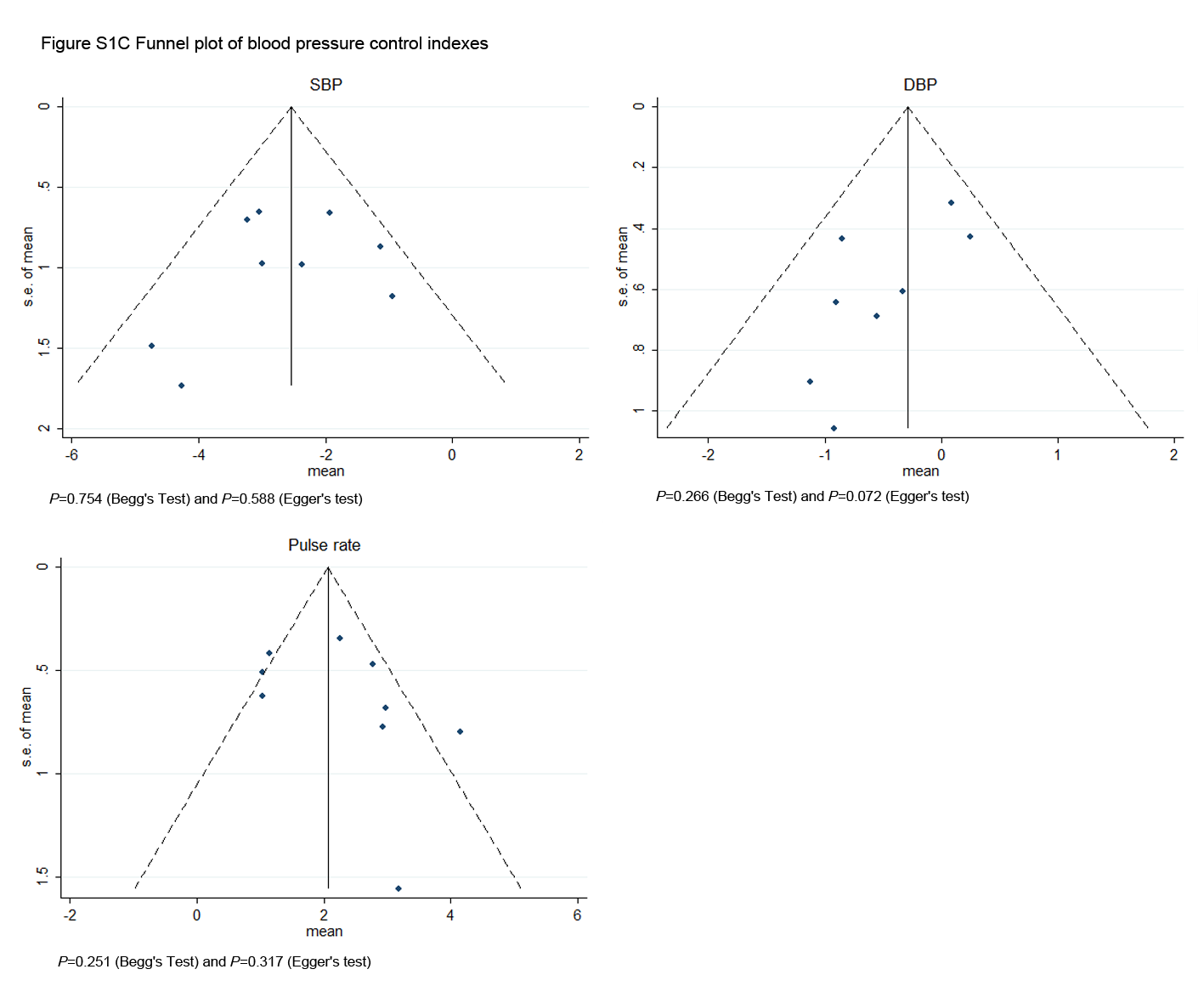

Supplement: Supplementary file 9 [file Image_3.tif]
